# A robot-based interception task to quantify upper limb impairments in proprioceptive and visual feedback after stroke

**DOI:** 10.1186/s12984-023-01262-0

**Published:** 2023-10-11

**Authors:** Kayne Park, Benjamin R. Ritsma, Sean P. Dukelow, Stephen H. Scott

**Affiliations:** 1 Centre for Neuroscience Studies, Queen’s University, Botterell Hall, 18 Stuart St, Kingston, ON K7L 3N6 Canada; 2 Department of Physical Medicine and Rehabilitation, Queen’s University, Kingston, ON Canada; 3 Department of Clinical Neurosciences, Hotchkiss Brain Institute, University of Calgary, Calgary, AB Canada; 4 Department of Biomedical and Molecular Sciences, Queen’s University, Kingston, ON Canada; 5 Department of Medicine, Queen’s University, Kingston, ON Canada; 6 Providence Care Hospital, Queen’s University, Kingston, ON Canada

**Keywords:** Proprioceptive feedback, Visual feedback, Stroke, Upper extremity, Reaction time, Robotic exoskeleton

## Abstract

**Background:**

A key motor skill is the ability to rapidly interact with our dynamic environment. Humans can generate goal-directed motor actions in response to sensory stimulus within ~ 60-200ms. This ability can be impaired after stroke, but most clinical tools lack any measures of rapid feedback processing. Reaching tasks have been used as a framework to quantify impairments in generating motor corrections for individuals with stroke. However, reaching may be inadequate as an assessment tool as repeated reaching can be fatiguing for individuals with stroke. Further, reaching requires many trials to be completed including trials with and without disturbances, and thus, exacerbate fatigue. Here, we describe a novel robotic task to quantify rapid feedback processing in healthy controls and compare this performance with individuals with stroke to (more) efficiently identify impairments in rapid feedback processing.

**Methods:**

We assessed a cohort of healthy controls (*n* = 135) and individuals with stroke (*n* = 40; Mean 41 days from stroke) in the Fast Feedback Interception Task (FFIT) using the Kinarm Exoskeleton robot. Participants were instructed to intercept a circular white target moving towards them with their hand represented as a virtual paddle. On some trials, the arm could be physically perturbed, the target or paddle could abruptly change location, or the target could change colour requiring the individual to now avoid the target.

**Results:**

Most participants with stroke were impaired in reaction time (85%) and end-point accuracy (83%) in at least one of the task conditions, most commonly with target or paddle shifts. Of note, this impairment was also evident in most individuals with stroke when performing the task using their unaffected arm (75%). Comparison with upper limb clinical measures identified moderate correlations with the FFIT.

**Conclusion:**

The FFIT was able to identify a high proportion of individuals with stroke as impaired in rapid feedback processing using either the affected or unaffected arms. The task allows many different types of feedback responses to be efficiently assessed in a short amount of time.

## Introduction

A key feature of our motor system is the ability to use sensory feedback for the online control of movement, such as responding when your arm is accidentally bumped when reaching for an object [1]. A broad range of studies highlight how proprioceptive and visual feedback can be used to generate goal-directed motor corrections and initiate new motor actions within 100ms [2, 3]. Impressively, even responses requiring cognitive functions such as aborting an on-going motor action can be generated within 200ms [4]. This use of proprioceptive and visual feedback for goal-directed motor actions is supported by highly distributed circuits including both cortical and subcortical structures [1, 5].

Previous studies highlight how stroke can impair an individual’s ability to generate rapid and accurate motor corrections [6–11]. Marsden et al. studied individuals with brain damage and identified delays in generating responses to mechanical perturbations applied during thumb movements [9]. Schaefer and colleagues examined responses to visual perturbations while participants reached towards a goal. They found individuals with stroke had delayed response initiation when compared to healthy controls [7]. Of note, ~ 30% of individuals with stroke display these impairments with their ‘unaffected’ arm [8, 12]. Identifying impaired corrective responses after stroke is crucial as reaction time impairments have been associated with reduced quality of life and an increased risk of falls [10, 13–16]. Importantly, the ability to use sensory feedback for motor function is not adequately considered in common clinical tools such as Fugl-Meyer Assessment (FMA) and Chedoke McMaster Stroke Assessment (CMSA) [17, 18].

The present study and associated behavioural task address several aspects of goal-directed feedback control. First, neural circuits associated with feedback processing involve a broad range of cortical and subcortical circuits. Thus, we hypothesize that impairments in using sensory feedback for goal-directed motor actions will be commonly observed in individuals following stroke. Second, neural pathways for proprioceptive and visual feedback overlap, but also involve distinct brain regions [19]. For example, primary somatosensory cortex and parietal area 5 are associated with somatosensory feedback, whereas occipital and posterior parietal regions are associated with visual feedback [20]. Thus, we hypothesize that individuals with stroke may display impairments that are limited to one sensory modality, proprioceptive or visual feedback. Finally, sensory feedback for motor and cognitive functions are also somewhat distinct, such as the involvement of medial regions in the frontal lobe engaged for inhibitory control [21]. Thus, we hypothesize that individuals with stroke may display selective impairment for either motor corrections or cognitive control.

We examined these hypotheses by developing a novel interception task that requires individuals to maintain their hand at a spatial location and were instructed to intercept a target moving rapidly towards them. On random trials, we induce mechanical or visual perturbations that require rapid corrective responses to intercept the moving target, or when instructed by a color cue, to avoid the target. The results highlight that most individuals with stroke are impaired in at least one form of feedback processing, and in some cases, selectively for one form of feedback processing.

## Methods

### Participants

Neurologically healthy control participants were recruited from Kingston, Ontario and surrounding region. Participants were included in the study if they were 18 years of age or older, were able to understand task instructions, and did not present with any neurological injury or disease, or ongoing musculoskeletal injuries impacting upper limb function. Participants with stroke were recruited from the inpatient stroke rehabilitation unit at Providence Care Hospital in Kingston, Ontario. Participants were included in the study if they had a confirmed unilateral stroke and were able to understand task instructions. Exclusionary criteria were substantial medical/neurological comorbidities (eg. Parkinson’s disease, multiple sclerosis), previous stroke, apraxia, or disease other than stroke affecting upper limb movement. This study has been reviewed by the Queen’s Health Sciences Research Ethics Board.

### Clinical measures

Participants with stroke were assessed by trained clinicians using common clinical measures. The National Institutes of Health Stroke Scale is an assessment of stroke severity [22]. The measure assesses many domains potentially affected by stroke (consciousness, sensory deficit, motor weakness) with a total score starting from 0 (no stroke deficits) with higher scores up to 42 indicating worse deficits. The Functional Independence Measure (FIM) examines the level of disability in various activities of daily living [23]. Our study used the motor component of the FIM (FIM-M) which is a composite score of 13 tasks such as grooming or eating, ranging from a score of 0 (no independence) to 7 (full independence) for a total score between 0 to 91. The Chedoke-McMaster Assessment Impairment Inventory: stage of arm and hand (CMSAa and CMSAh, respectively) examined the ability to use the arm and hand in upper extremity movements where scores ranged from 0 (flaccid arm/hand) to 7 (normal timing, co-ordination, strength and endurance) [18]. The Montreal Cognitive Assessment (MoCA) is a screening tool assessing the presence of mild cognitive impairment where scores (0–30) below 27 indicate potential impairment [24]. The Behavioral Inattention Test (BITC) Conventional Subtest assesses the presence of visuospatial neglect on tasks such as cancellation tasks and figure copying [25]. Out of a possible 146, scores below 130 indicate the presence of neglect. Clinical examinations of individuals with stroke also determined one side of the body as being hemiparetic. We denote the side with hemiparesis as “affected” and the other side as “unaffected” with the understanding that impairment has been revealed even in the “unaffected” side post-stroke [26]. Further, we refer to individuals presenting with left hemiparesis as Left Affected (LA) and those with right hemiparesis as Right Affected (RA).

### Robotic setup

Experiments were conducted using the Kinarm Exoskeleton Lab (Kinarm, Kingston, Ontario) [27]. The Kinarm robot quantifies kinematic and kinetic performance of the upper limb, described in detail previously [8]. Briefly, participants were seated in an adjustable height wheelchair with each arm supported against gravity by plastic troughs attached to mechanical linkages that permit arm movements in the horizontal plane. Motors attached to the linkage measure arm motion and can apply loads to the limb, as required. The system includes an integrated virtual reality system that displays visual objects and feedback of hand position aligned with the horizontal workspace. During experiments, vision of the upper limbs was occluded with a physical barrier.

### Robotic assessment

**Fig. 1:**
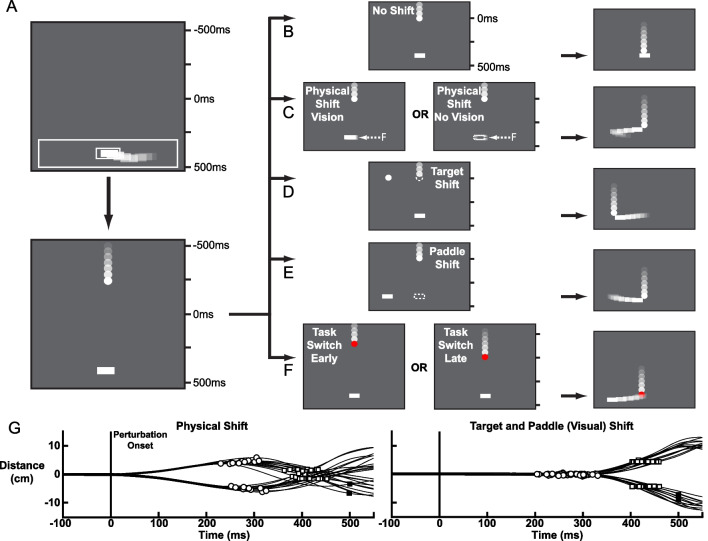
FFIT paradigm layout. **A** Start of each trial. Top portion presents the paddle (hand represented as a white rectangle) moving to the small open rectangle (start target) inside of a large open rectangle (Active Region). The increase in transparency (after images) of the paddle and targets in these plots represent movement but were not displayed during the task. The bottom portion displays the movement of the target. Start target and Active Region were visually removed but physical restrictions of the Active Region were still engaged. **B**–**F** The left column displays the perturbation onset for No Shift (**B**), Physical Shift (**C**), Target Shift (**D**), Paddle Shift (**E**), Task Switch (**F**) trials. The right column highlights corrective motor responses for each respective trial. Dashed targets were not present during the task but signify rapid spatial changes. **B** No Shift trial where no perturbation was applied, and participants only held their posture. **C** Physical Shift trial type where a physical perturbation was applied to participants arms towards the left or right (50%) and vision of the paddle was on or off (50%). **D** Target Shift trial consisted of a rapid shift in the position of the target (50% left or right jump). **E** Paddle Shift trial consisted of a rapid shift in paddle location (50% left or right jump). **F** Task Switch trials changed the ball colour from white to red indicating the novel rule to avoid the target. These trials were split into early (-200ms) or late (-50ms) timepoints. **G** Plots displaying distance over time for the hand of an exemplar participant for Physical Shift (left panel) and Target and Paddle Shift trials (right panel). Perturbation onset at 0ms (solid line), circles indicate Reaction Time, open squares represent successful contact between paddle and target, and filled squares represent unsuccessful trials

We developed a task called the Fast Feedback Interception Task (FFIT; Fig. 1A–F). The main goal of the task is for participants to use a white paddle (rectangle of width = 2cm, length = 0.5cm) aligned with their index finger to intercept a moving white target (circle of radius = 0.56cm; velocity 25cm/s). Hand movements were physically restricted by mechanical forces applied by the robot to stay within an “Active Region" (28cm wide by 5cm length) centered on the central start position. This was done to limit where the individual could use the paddle to intercept the target. Each trial began with participants holding their hand at a central location (30° shoulder adduction and 90° elbow flexion) for 250ms. After this period, the white target would appear at the top of the workspace and start moving directly towards the bottom of the workspace. On 20% of trials the target would move towards the bottom of the workspace and the participant would simply stay at the central start position until the target was intercepted by the paddle (1B baseline trials in Fig. 1). On the remaining 80% of trials, after the target started moving towards the participant (500ms), one of four perturbations would occur at equal probabilities. For one trial type (Fig. 1C), the robot applied an ~ 6N effective load to the hand (Physical Shift trials) deviating the hand to the left or right at equal probability. In half of these trials, visual feedback of the paddle was removed so that motor corrections relied on proprioceptive feedback. Participants were required to counteract this load to successfully intercept the target. These trials quantified the ability of individuals to make goal-directed motor corrections to mechanical disturbances of the limb [1]. In a second type of trial (Fig. 1D), the moving target was displaced 6cm to the left or right, and then continued moving towards the bottom of the workspace (Target Shift trials). Participants would be required to generate a left or right movement to successfully intercept the target. In another similar trial type (Fig. 1E), the location of the paddle rather than the target was displaced 6cm left or right (Paddle Shift trials) while the target continued to move down. These trials quantified the ability of individuals to make goal-directed motor corrections to visual disturbances related to the arm or behavioural goal [1]. Participants would need to move their displaced paddle to the center of the Active Region to intercept the target. For the final trial type (Fig. 1F), the target switched colour from white to red. Participants were previously instructed that this change of target colour indicated a new task goal to avoid rather than intercept the target (Task Switch trials). These trials quantified the ability of individuals to use sensory feedback or information to abort an ongoing motor action [1]. For the Target Shift, Paddle Shift, and Physical Shift trials, the participant could intercept the target as it passed through the Active Region between 300 and 500ms after perturbation onset. For the Task Switch trials, the color changes 300ms (early) or 450ms (late) after the target started moving. This resulted in the moving target passing through the Active Region 500-700ms (early) or 350-550ms (late), respectively, after the colour change. Contact between the paddle and target was simulated based on contact speed and hand angle by haptic feedback applied to the arm. On Task Switch trials, any unsuccessful target avoidances would not apply haptic feedback and the target would pass through the paddle to remind the participant they were meant to avoid the target on these trials. The trial would end when the target passed the Active Region (unsuccessful trial) or 500ms after contact (successful trial). There was an inter-trial interval of 500ms before the start of the next trial.

There was a total of 120 trials with an equal proportion of each trial type (24 trials each). The task was organized into 3 blocks of 20 trials (4 of each trial type) randomly interleaved and then repeated once (3 × 20×2). Participants were initially given 6 trials (No Shift, Target Shift, Paddle Shift, Physical Shift, Physical Shift (no paddle vision), Task Switch (early)) to familiarize themselves with the basic goal of the task and types of disturbances that may occur. Each arm was assessed separately, and the order was chosen randomly. Data for a given arm was not analyzed if participants did not complete at least 2 trials in each direction (left or right) of each trial type.

### Performance measures

The objective of the task is to assess one’s ability to make fast and accurate motor corrections. Our most important measure was the speed of processing sensory feedback related to the arm and spatial goals. Reaction Time for perturbations was calculated as the difference in time from perturbation onset to movement onset. Movement onset differed based on the trial type. In No Shift trials, no perturbation was applied, and thus, there is no movement onset or Reaction Time. For Target Shift, Paddle Shift, and Task Switch trials, movement onset was based on an increase in lateral hand speed above what was observed while participants kept the paddle at the central start location (postural speed: mean speed 500ms prior to perturbation), using techniques previously described to identify the initiation of reaching [28]. Briefly, once the participant leaves the start position, the algorithm moves back in time until either (1) a local minimum in hand speed occurs below 95% of their postural speed or (2) hand speed was below the median postural speed. Although the target passed through the Active Region 500ms after perturbation onset (550ms and 700ms for Task Switch trials), corrective responses would sometimes be elicited after the target disappeared. Thus, the maximum allotted time for responses included the inter-trial period of 500ms for a total of 1000ms. In trials where the hand speed did not reach the median postural speed (i.e. did not move), Reaction Time was set to this maximum allotted time of 1000ms.

Movement onset differed for Physical Shift trials as the mechanical load generates motion of the limb. A method to determine movement onset to mechanical perturbations has been described previously [12]. In brief, hand speeds during mechanical loads commonly display an initial peak in speed which then decreases with time to a minimum value as participants counteract the load and start to return to their initial position. This minimum in hand speed (deceleration time) is our definition of movement onset for Physical Shift trials. This time point usually corresponds to the maximum displacement of the hand. For trials where individuals were unable to counteract the mechanical load, resulting in a large displacement of the hand and contact on either the left or right side of the Action Region, Reaction Time was set to the maximum allotted time of 1000ms. We refer to movement onset for Physical Shift trials as Reaction Time to simplify parameter names in this study but understand that response time is a more accurate description.

We also quantified the spatial accuracy of the motor corrections. Participant’s accuracy in this task, termed Endpoint Distance, was measured simply by the closest distance between the center of the target and the center of the paddle at either (1) the time when the target and paddle contact or, if there was no contact, (2) the closest distance between target and paddle as the target passed through the Active Region. This distance was not adjusted for the target radius or the paddle width and length. Endpoint Distance closer to 0cm indicated better performance for all trials except for Task Switch trials where greater values indicated better performance.

### Data analysis

Robotic data were sampled at a rate of 1000Hz and collected using the Dexterit-E software program (v3.6, 3.7, and 3.8, Kinarm, Kingston, Ontario). Data was filtered using a sixth-order double-band pass filter with a cutoff frequency of 10Hz and analyzed using Matlab 2018b (Mathworks Inc., Natick, Massachusetts).

Statistical analyses were performed on Matlab (Mathworks Inc., Natick, MA, USA). To identify potential impairments in the use of sensory feedback, we first need to identify healthy performance. Normative models of control participant’s Reaction Time and Endpoint Distance were developed to account for the effects of age, sex, and handedness [29]. These parameters were transformed into standard normal distributions using Box-Cox Equations [30] which were checked for normality with a Shapiro-Wilkes Test. Healthy controls with Z-scores greater than 3.29 or less than -3.29 were removed as outliers with the entire process repeated up to three times or until there were no more outliers. The models created from these distributions were then applied to each individual with stroke. The healthy range of performance in each measure was set at a Z-score of 1.65 (95% Confidence Interval (CI)) and stroke individuals with Z-scores greater than this value were identified as impaired.

Next, to identify whether the “affected side” of the body had an effect on behavioural measures, we compared the FFIT Reaction Time, Endpoint Distance, and clinical scores of LA individuals to RA individuals (e.g. NIHSS, BITC, etc.…) using a Kolmogorov-Smirnoff (K-S) Test. We sought to identify whether affected or unaffected arm FFIT performance was significantly different using a paired T-Tests. To understand the relationship between task performance and clinical measures, we calculated Spearman’s correlation coefficient between normalized Reaction Time/Endpoint Distance and clinical scores.

## Results

Demographic information for the 40 individuals with stroke and 135 control participants is shown in Table 1. Clinical information of the LA and RA individuals was largely similar although there was a tendency for LA individuals to be more impaired. A greater number of LA participants had low CMSAa scores (score < 3; no voluntary activity) for the affected arms (*n* = 7) compared to RA participants (*n* = 4). As well, there were minor non-significant differences in for NIHSS (*p* = 0.95), FIM (*p* = 0.95), MOCA (*p* = 0.55), and BITC (*p* = 0.50) scores between LA and RA individuals.

**Table 1:** Demographic Information of control and stroke participants

	Controls (n = 135)	Left affected (LA) individuals (n = 20)	Right affected (RA) individuals (n = 20)
Age (Min, 25%, 75%, Max)	40 (18, 22, 59, 83)	65 (42, 59, 75. 84)	69 (46, 63, 77, 87)
Sex (M/F)	58/77	9/11	10/10
Handedness (L/R)	17/118	2/18	1/19
Stroke Type (H/I/U)		4/12/2	8/11/1
Days Since Stroke (Min, 25%, 75%, Max)		47 (9, 24, 57, 129)	34 (11, 23, 31, 88)
CMSA Affected Arm [1/2/3/4/5/6/7]		[5/2/2/2/3/1/4]	[2/2/7/2/1/2/3]
CMSA Unaffected Arm		[0/0/0/0/3/7/9]	[0/0/0/0/3/7/9]
CMSA Affected Hand		[3/4/0/2/5/4/1]	[1/2/3/1/6/5/1]
CMSA Unaffected Hand		[0/0/0/0/2/5/12]	[0/0/0/0/0/7/12]
NIHSS (Min, 25%, 75%, Max)		5 (0, 2, 8, 11)	3 (0, 2, 5, 10)
FIM-M (Min, 25%, 75%, Max)		65 (38, 47, 79, 86)	72 (48, 66, 81, 91)
MOCA (Min, 25%, 75%, Max)		24 (18, 22, 27, 30)	24 (19, 22, 26, 28)
BITC (Min, 25%, 75%, Max)		137 (117, 130, 145, 146)	142 (121, 141, 144, 146)
Stroke Location [C/SC/C + SC/Ce/Br/Ce + Br/U]		[8/5/4/1/0/0/0]	[5/12/1/1/1/0/0]

Demographic and clinical information for healthy controls and individuals with stroke separated into left and right affected stroke. Age, Days since stroke, NIHSS, FIM-M, MoCA, BITC are displayed as mean with the minimum, maximum, and first and third interquartile below. Sex, Handedness, Stroke type, CMSA, Stroke location are shown as values split according to the subtypes identified below:

- Sex [Male/Female]
- Handedness [Left/Right/Ambidextrous]
- Stroke type [Ischemic/Hemorrhagic/Unknown]
- CMSA Scores of [1/2/3/4/5/6/7]
- Stroke Location [Cortical/Subcortical/Cortical + Subcortical/Cerebellar/Brainstem/Cerebellar + Brainstem/Unknown]

There were roughly equal numbers of LA and RA participants who were able to perform the interception task. However, some individuals with stroke were unable to maintain their hand at the start position with their affected arm, and thus, performance in the task could not be assessed (LA, *n* = 5, average CMSAa = 2.2; RA, *n* = 2, average CMSAa = 2). There were less individuals with stroke unable to perform the FFIT than were able to perform voluntary actions (CMSAa score < 3). This is unlikely attributable to time differences between robot-based and clinical assessment which was only 1.7 days. A likely reason for this difference is the gravity support provided by the Kinarm Exoskeleton.

### Individual FFIT performance

#### Exemplar performance on no shift trials

**Fig. 2:**
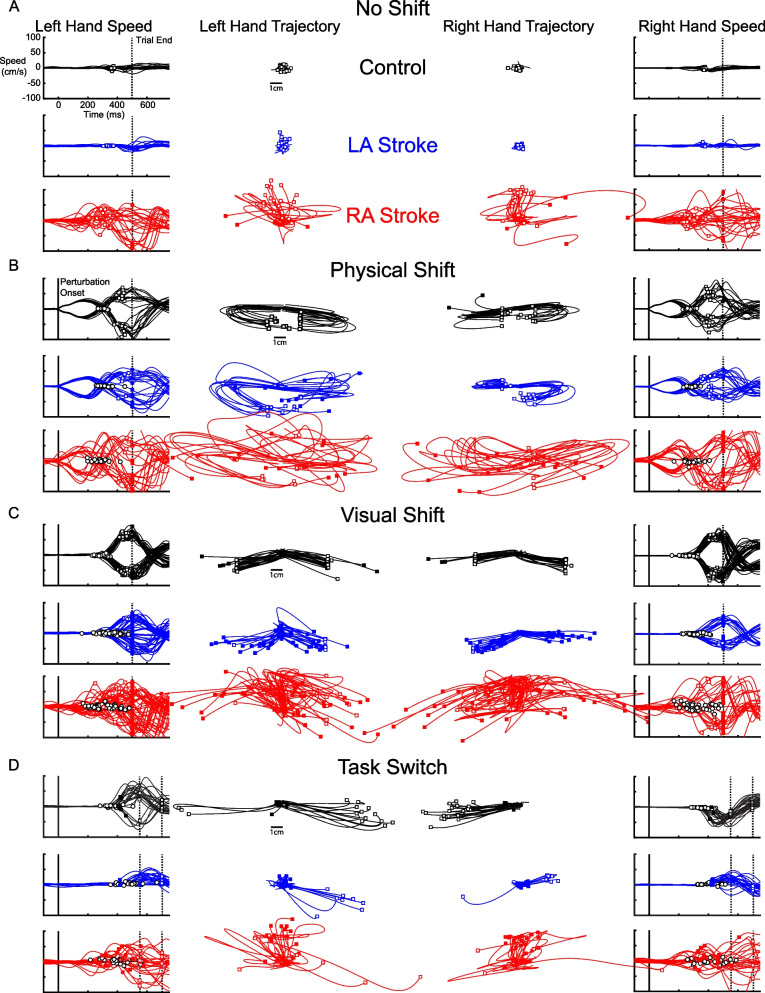
Hand speeds and trajectories for an exemplar healthy control and participants with stroke during the FFIT. **A** Hand speed and trajectories for No Shift trials for a healthy control in black, left affected individual in blue, and right affected individual in red. Hand speeds are plotted with 0ms aligned to perturbation onset and 500ms at trial end represented with a dashed line. Hand trajectories are plotted from perturbation onset (0ms) to time of contact between paddle and target (open squares) or trial end (missed contact; filled squares). **B** Physical Shift trials for the exemplar participants where perturbation onset in hand speed plots is shown with a solid vertical line. Reaction Time is displayed as open circles. **C** Visual Shift trials combining both Target Shift and Paddle Shift trials for exemplars. **D** Task Switch trial performance for exemplars. Two dashed lines are shown to depict the Early and Late variants for this trial type. The Early Task Switch trials end at 700ms and Late end at 550ms

Figure 2 displays hand trajectories and corresponding hand speeds for a neurologically healthy exemplar (in black), LA participant (in blue), and RA participant (in red). These plots highlight the speed (outer panels) and location (inner panels) of the participant’s movements. Figure 2A displays performance for the exemplar participants in the No Shift trials which required the individual to simply maintain their hand at the starting position to successfully intercept the target. As expected, the neurologically healthy control participant displayed minimal hand motion. The LA individual also displayed minimal hand motion for these trials. In contrast, hand motion was much larger for the RA individual, demonstrating their difficulty in holding a static position with both their left and right arms.

#### Exemplar performance on physical shift trials

Figure 2B highlights exemplar performance in Physical Shift trials. We found participants performed similarly whether or not visual feedback of the paddle was removed (*r* = 0.89, *p* < 0.01), and thus, our analysis grouped these trials together. The load moved the hand away from the central start location and the individual quickly returned their hand to the center to intercept the moving target. Speed profiles and hand trajectories were highly consistent across trials for the healthy control. The LA participant was able to generate responses but performed generally worse than the control participant. Their hand speeds and trajectories show consistent movements, but relative to the control, they displayed a wider distribution of Reaction Times and Endpoint Distances using both affected and unaffected arms. The RA exemplar showed large trial-to-trial variability in Physical Shift trials for both arms. Although they clearly generated responses to the mechanical perturbations, they were highly variable across the trials resulting in inconsistent Reaction Times and Endpoint Distances.

#### Exemplar performance on visual shift trials

We found participants with stroke performed similarly for Target and Paddle shift trials (*r* = 0.83, *p* < 0.01), and thus, these two trial types were grouped into one trial type termed Visual Shift trials. Visual Shift trials for the exemplars are shown in Fig. 2C. The control participant demonstrated distinct goal-directed movements and consistent Reaction Times and Endpoint Distances. The LA individual’s responses were less consistent than the exemplar control which was demonstrated by greater variability in Reaction Time and Endpoint Distance. The RA individual had clear difficulties responding to Visual Shift trials. In both arms, left and rightward movements are difficult to distinguish and their Reaction Times and Endpoint Distance were highly variable.

#### Exemplar performance on task switch trials

Finally, Task Switch trials are displayed in Fig. 2D. As the objective was to avoid the target, successful trials required hand movements away from the central location. The control participant was able to avoid the target for most trials. One can see that the control participant had greater Reaction Time variability in Task Switch trials than other trial types, highlighting the difficulty to abort the ongoing motor action of intercepting the moving target. The LA individual had difficulty responding to the stimulus change in either arm as they were unable to avoid the target in most trials. The RA individual also performed poorly for most trials, although their success in a low proportion of trials may be due to their difficulty in holding posture rather than their ability to rapidly alter their motor plan to avoid red targets.

### Group performance

#### Impairments associated with LA and RA individuals

Table 2 displays the percentage of individuals with stroke that were impaired in Reaction Time and Endpoint Distance after controlling for age, sex, and handedness (individuals unable to perform the task were identified as impaired in each measure; LA, *n* = 7; RA, *n* = 2). A large proportion of LA individuals were impaired on Reaction Time. Using the affected arm, 85% of all LA individuals were impaired in at least one trial type with 62% impaired in Physical Shift, 69% impaired in Visual Shift, and 62% impaired in Task Switch trials. Many RA individuals, using their affected arm, were identified as impaired with 83% impaired across all trials, 67% in Physical Shift, 78% in Visual Shift, and 56% in Task Switch trials. Interestingly, RT between the LA and RA groups were not significantly different from each other (Physical Shift, *p* = 0.25; Visual Shift, *p* = 0.72; Task Switch, *p* = 0.82).

**Table 2:** Impairment for transformed FFIT parameters for LA and RA participants

Reaction time	Total impaired (%)	Physical shift impaired (%)	Visual shift impaired (%)	Task switch impaired (%)
LA stroke				
Left arm	85	62	69	62
Right arm	60	40	55	45
RA stroke				
Left arm	55	15	40	40
Right arm	83	67	78	56

Endpoint distance	Total impaired (%)	Physical shift impaired (%)	Visual shift impaired (%)	Task switch impaired (%)
LA stroke				
Left arm	77	69	69	31
Right arm	75	55	70	35
RA stroke				
Left arm	50	30	40	20
Right arm	83	78	72	39

% impairment for LA and RA individuals across all trial types for Reaction Time and Endpoint Distance

Importantly, impairments were commonly observed in the ‘unaffected’ arm despite only 12.5% of individuals with stroke scoring < 6 on the CMSAa (full range of motion with near normal timing and coordination). We found 60% of LA individuals were impaired in their unaffected arm, with 40% identified as impaired in Physical Shift trials, 55% in Visual Shift trials, and 45% in Task Switch trials. For RA individuals, 55% were impaired using their unaffected arm, including 15% in Physical Shift trials, 40% in Visual Shift trials, and 40% in Task Switch trials. Unaffected arm RT for LA and RA individuals were not significantly different (Physical Shift, *p* = 0.06; Visual Shift, *p* = 0.50; Task Switch, *p* = 0.50).

On some trials, we did not identify a Reaction Time following the perturbation. In these trials we set Reaction Time to the maximum allotted time (1000ms). For LA individuals, the mean percentage of trials set to 1000ms was 23% (range = 2–46%) for the affected arm, and 11% (range 1–39%) for the unaffected arm. For RA individuals, the mean for the affected arm was 15% (range = 0–60%) and the unaffected arm was 4% (range = 0–10%). Mechanical disturbances were associated with the greatest percentage of trials without an identified Reaction Time (mean = 37% across all individuals and arms). In comparison, we did not identify a Reaction Time for only 3% of trials for healthy control participants.

Much like Reaction Time, impairments were commonly observed for Endpoint Distance across all trial types and for both the affected and unaffected arms. We identified 77% of LA participants with impaired Endpoint Distance in their affected arm for at least one trial type (69% in Physical Shift, 69% in Visual Shift, and 31% in Task Switch trials). For RA individuals, 83% were impaired in Endpoint Distance for the affected arm for at least one trial type (78% in Physical Shift trials, 72% in Visual Shift trials, and 39% in Task Switch trials). The Endpoint Distance for the affected arm of LA and RA groups were not significantly different (Physical Shift, *p* = 0.51; Visual Shift, *p* = 0.57; Task Switch, *p* = 0.88).

Impairment in Endpoint Distance was also common in the unaffected arms. For LA individuals, 75% were impaired in at least one trial type, with 55% in Physical Shift, 70% in Visual Shift, and 35% in Task Switch trials. For RA individuals, 50% were identified as impaired across all trial types, with 30% impaired in Physical Shift, 40% in Visual Shift, and 20% in Task Switch trials. Only Task Switch trials were significantly different between LA and RA individuals using their unaffected arm (Physical Shift, *p* = 0.13; Task Switch, *p* = 0.28; Task Switch, *p* < 0.05).

#### Comparison of impairments associated with the affected and unaffected arms

**Fig. 3:**
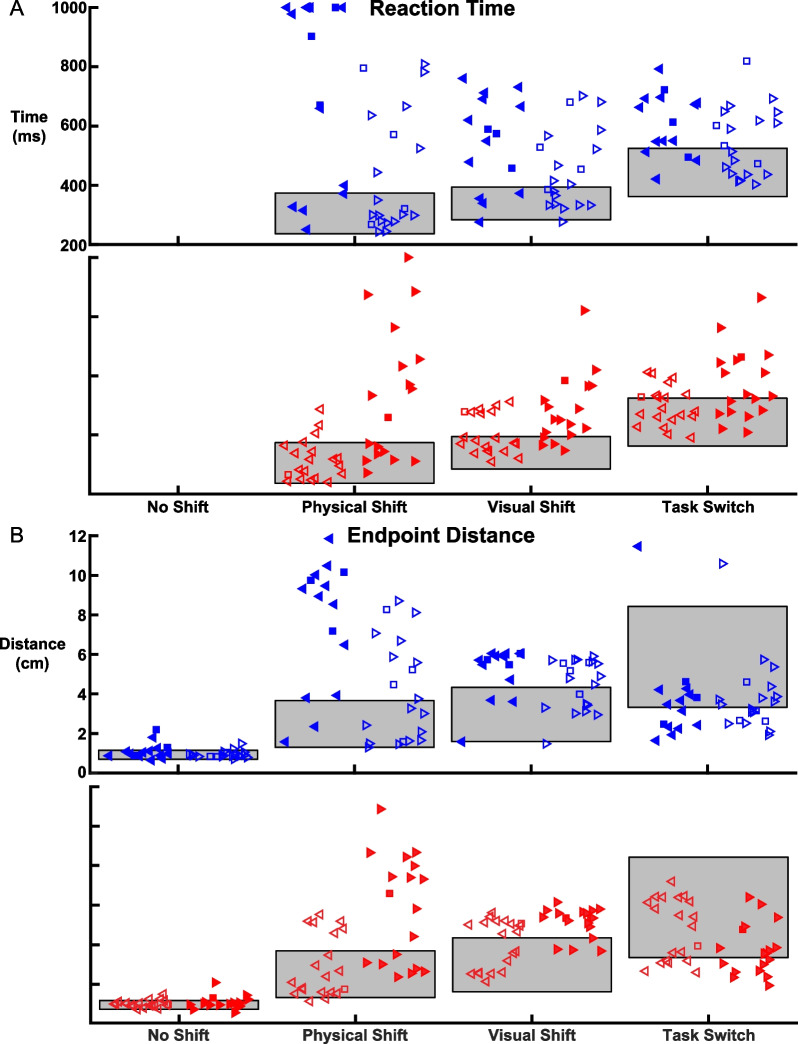
Group level Reaction Time and Endpoint Distance performance across trial types.** A** The Reaction Time of LA individuals (blue; top panel) for both their left (filled left-directed triangles) and right arms (unfilled right-directed triangles). Individuals that failed the BITC (presenting with visuospatial neglect) are identified as square icons. The healthy control performance in each trial type is plotted as a grey rectangle where the top and bottom of the rectangle represent the 95 and 5 percentiles of Reaction Time, respectively. Individuals with stroke were identified as impaired if their Reaction Time was greater than 95% of controls. The bottom panel presents the Reaction Time of RA individuals (red) with the left arm performance as unfilled left-directed triangles and the right arm as filled right-directed triangles. **B** Endpoint Distance of healthy controls, LA, and RA individuals in the same format as (**A**). Impairment in Endpoint Distance was identified as being greater than 95% of controls for No Shift, Physical Shift, and Visual Shift trials. Impaired Endpoint Distance for Task Switch trials was characterized by Endpoint Distance less than 5% of controls

Figure 3 displays the untransformed Reaction Time and Endpoint Distance across our cohort of 135 controls and 40 individuals with stroke. Reaction Time performance is plotted in Fig. 3A. For control performance, one can see an increase in Reaction Time where Physical Shift trials elicit the fastest Reaction Time, Visual Shift trials elicit slower times, and Task Switch trials had the slowest times. For LA individuals, Physical Shift trials displayed a wide range of impairment with some individuals reaching the maximum allotted Reaction Time. The affected arm was significantly more impaired than the unaffected arm for Physical Shift trials (*p* < 0.01) but not for Visual Shift or Task Switch trials. The bottom panel displays Reaction Time of controls and RA individuals. These individuals displayed significantly greater impairment in RT for the affected rather than unaffected arm for Physical (*p* < 0.01), Visual (*p* < 0.05), and Task Switch (*p* < 0.05) trials.

Endpoint Distance is displayed in Fig. 3B. We identified 4 LA individuals impaired in No Shift trials, using their affected arm, highlighting an impairment in maintaining the hand at a spatial location. Of note, Endpoint Distance in Visual Shift trials was commonly impaired as most LA individuals had an Endpoint Distance near 6cm which is the maximum distance between paddle and target after the visual perturbation. Endpoint Distance was significantly more impaired for the affected arm compared to the unaffected arm for Physical (*p* < 0.01) and Visual Shifts (*p* < 0.01). The RA individuals in the bottom panel performed similarly to the LA individuals with significantly more impairment for all trial types using the affected arm rather than unaffected arm, except for No Shift trials (Physical, *p* < 0.01; Visual, *p* < 0.01; Task Switch, *p* < 0.05).

#### Impairments associated with different sensory modalities

**Fig. 4:**
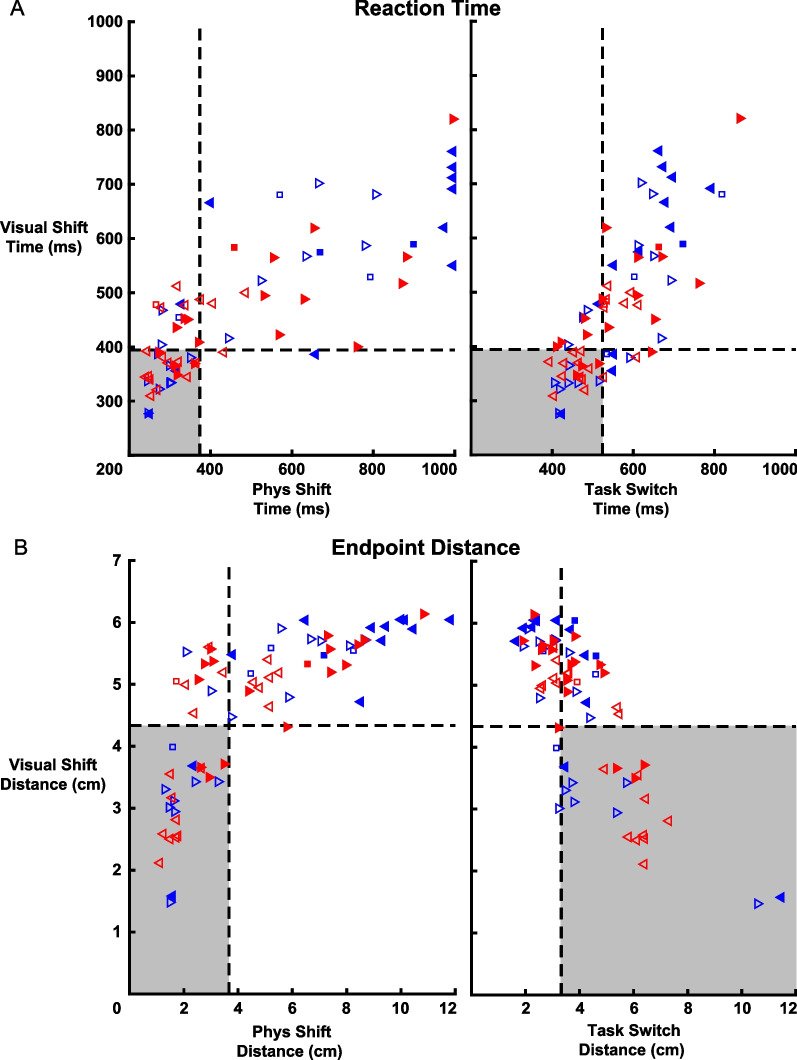
Group level comparison between trial types. **A** Comparison of trial types for Reaction Time. The left panel displays Reaction Time for the Physical Shift and Visual Shift trial types with triangle colour and direction denoting affected arm and assessed arm, respectively, in the same format as Fig. 3. The healthy control region is displayed as a grey box with dashed lines representing the 95 percentile of control performance. The right panel shows the comparison between Visual Shift and Task Switch trials. **B** Trial type comparison for Endpoint Distance. The left panel compares Endpoint Distance for Physical Shift and Visual Shift trials in the same format as the left panel in (**A**). The right panel compares Visual Shift and Task Switch trials where impaired Visual Shift Endpoint Distance is greater than 95% of controls but impairment for Task Switch trials was identified as performance less than 5% of controls

To identify impairments specific to one sensory system, we compared Reaction Time between different trial types (Fig. 4). The left panel of Fig. 4A displays the Reaction Time impairment for LA and RA individuals in Physical and Visual Shift trials. In both Physical and Visual Shift trials, 15 (71%) and 8 (38%) LA individuals were using their affected and unaffected arms, respectively. In contrast, 10 (53%) RA individuals were impaired in both trial types in the affected arm, whereas only 3 (16%) were impaired in the unaffected arm. There were 3 LA individuals and 4 RA individuals displaying Reaction Time impairment in visual but not proprioceptive feedback using their unaffected arm. The right panel of Fig. 4A compared Visual Shift and Task Switch trials. Again, most individuals were impaired in both trial types and only a few individuals impaired in only Visual Shift or Task Switch trials. Figure 4B highlights the same comparisons as in Fig. 4A but for Endpoint Distance. For both left and right panels, performance in Endpoint Distance also displayed similar patterns of impairments with the majority impaired in both trial types. This reflects the high correlation between Endpoint Distance and Reaction Time in each trial type (Physical Shift: *r* = 0.95, *p* < 0.01; Visual Shift: *r* = 0.91, *p* < 0.01; Task Switch: *r* = − 0.77, *p* < 0.01). Interestingly, there were many individuals with Endpoint Distance impairment in only Visual Shift trials and not Task Switch Trials (LA, *n* = 5; RA, *n* = 7).

#### Comparison of FFIT performance and clinical measures

To test the relationship between FFIT performance and the clinical measure scores of individuals with stroke, we performed Spearman rank-order correlations. For LA individuals, we found moderate correlations between Reaction Time impairment and CMSAa scores for Physical Shift (*r* =− 0.70, *p* < 0.05 RA participants had Reaction Time impairment that was moderately correlated with CMSAa scores for Visual Shift trials (*r* = − 0.53, *p* < 0.05) and Task Switch trials (*r* =− 0.52, *p* < 0.05). We identified no significant correlations between Reaction Time impairment with the MoCA or FIM-M and only near significant correlations with the BITC (Visual Shift trials: *r* =− 0.67, *p* = 0.055).

Endpoint Distance impairment was significantly correlated with the CMSAa. For LA individuals, Physical (*r* = − 0.69, *p* < 0.01) and Visual Shift (*r* = − 0.62, *p* < 0.05) trials had moderate correlations between Endpoint Distance and CMSAa. RA individuals only had moderately significant correlations between CMSAa and Physical Shift trials (*r* = − 0.60, *p* < 0.05). Further, we noted 4 individuals with stroke had CMSAa scores of 6 and 7 (full range of motion with near normal timing and coordination) but were still identified as impaired in Reaction Time and Endpoint Distance.

## Discussion

The present study used a novel postural interception task to assess the ability of individuals with stroke to generate rapid motor corrections. Reaction Time and Endpoint Distance measures were able to identify as many as 85% of LA individuals and 83% of RA individuals with impairments using their affected arms. Importantly, over half of individuals with stroke were impaired with their unaffected arm. Impairments associated with different types of visual feedback were highly overlapping, and some individuals displayed distinct impairments for either proprioceptive or visual feedback responses. We found impairments in Reaction Time and Endpoint Distance significantly correlated with clinical measures of upper limb impairment (CMSAa), but not with measures of functional independence (FIM-M), cognitive testing (MoCA), or visuospatial neglect (BITC).

### Rapid feedback as a clinical tool

Here, we introduce FFIT as a paradigm that can provide an objective assessment for the use of sensory feedback to generate motor actions. The task requires about 6 min to complete and assesses many different types of sensory feedback processing. Our key finding was that 85% of individuals with stroke were impaired when compared to a large cohort of healthy controls. Previous reports of sensory impairments related to stroke range from 44 to 85% [31–34]. These clinical assessments focus on impairments in sensory perception whereas the FFIT has been designed to assess impairments in the use of sensory feedback for action. This is an important distinction to make as these two uses of sensory feedback have been demonstrated to have dissociable neural pathways and behaviour [35, 36]. Further, we found impairments in generating motor corrections were modestly correlated with clinical measures of motor impairment highlighting the obvious relationship between sensory feedback and motor actions, an avenue for future research.

The importance for identifying impairments in sensory feedback is highlighted by studies examining fall risk. Previous work by Maki and McIlroy examined how the upper limb can be used to grasp a handrail to counter lower limb disturbances during stance [37]. They identified similar timing of muscle activity in the upper and lower limbs in response to postural perturbations highlighting highly coordinated whole-body responses to maintain balance. Further, these individuals may have a general impairment in generating motor corrections and thus also lack rapid motor responses by the lower limbs. Our results highlight the one of many potential influences for increased fall risk. Thus, a topic for future studies is to understand the relationship between impaired use of sensory feedback in the upper and lower limbs and fall risk [15, 38, 39].

### Rapid sensory feedback after stroke

An advantage of the presented paradigm is that we can easily contrast and compare motor impairments associated with different feedback processes and neural pathways. Our results illustrate that most individuals with stroke have difficulty generating motor corrections for both proprioceptive and visual feedback. However, we also found a select few individuals with stroke that displayed impairments in visual and not proprioceptive feedback. This may be because the onset of the motor response is fairly easy to identify for visual disturbances, whereas the mechanical load moves the limb away and we could only identify time when the hand reverses direction. Interestingly, we were unable to find many individuals with stroke impaired in Task Switch trials and not impaired in Visual Shift trials or vice versa. This result may have been influenced by the greater allotted time allowed to respond to Task Switch trials but requires further investigation.

This greater predominance for impairments related to visual disturbances may reflect differences in the neural pathways associated with proprioceptive and visual feedback. Goal-directed motor corrections to mechanical disturbances involve transcortical pathways including parietal and motor cortical regions [20, 40]. In contrast, visual feedback processing involves these frontoparietal circuits as well as occipital and broader parietal regions implicated in visuomotor actions [41–43]. Visual feedback impairment due to a parietal lesion has been highlighted by Gréa and colleagues [44]. An individual with a bilateral posterior parietal cortex lesion performed a reaching movement to a cylinder that could rapidly shift location during the reach. Reach initiation towards the cylinder was initiated at a similar time as healthy controls but the individual was delayed in responding to the visual perturbations. This demonstrates the relationship between lesion location and sensory feedback impairment but is focused on visual feedback. Previous studies highlight that lesions to posterior parietal cortex, primary somatosensory cortex, and arcuate fasciculus commonly lead to impairments in proprioception, the use of limb sensory feedback for perception [45–47]. How sensory feedback impairments in the FFIT are related to the size and location of the stroke and impairments in the use of sensory perception are an important issues we hope to address in the future.

We were able to simplify our task analysis as Physical Shift trials with and without vision were performed similarly. Both trial types were assessed to identify the potential influence that visual feedback from the cursor could have on proprioceptive feedback processing. Our findings are in-line with previous work highlighting only a modest contribution of visual feedback for rapid motor actions [48]. Further, we combined Target and Paddle Shift trials into Visual Shift trials as they also were performed similarly. Interestingly, our data (not shown) found that Target Shift trials were generated faster than Paddle Shift trials (~ 40ms). This is particularly interesting as others have found that responses to cursor perturbations have been elicited faster than target perturbations [49]. A likely reason for this may be due to the difference in generating responses during reaching and interception.

A key finding in the present study was a large proportion of individuals presenting with impairment associated with both arms. Clearly, stroke is generally associated with impairments of the contralesional side of the body [50]. Some studies highlight impairments associated with the ipsilesional side although these impairments are generally less severe than the contralesional side [26, 28, 51, 52]. Previous studies have noted that both limbs can be impaired for motor corrections to mechanical loads during postural control [8, 12]. Here, we demonstrate that visual-based motor corrections for both limbs can also be impaired. This suggests that the ability to generate motor corrections are a bilateral process and may be related to observations that many neurons in motor cortex are related to both limbs including responses to mechanical disturbances of either limb [53–55].

## Limitations

There is substantial evidence suggesting an association with left hemispheric damage and aphasia. Our study excluded individuals who were unable to understand task instructions and, thus, potential recruitment of RA individuals was reduced.

For some of our individuals with stroke, impairment in the affected arm was severe enough that they were unable to maintain their hand at the central location (LA, *n* = 7; RA, *n* = 2). We identified these individuals as impaired in the task as attaining the central location in itself requires feedback corrections. However, the lack of completing any trials means that there is a small floor effect in this task that impacts assessing the most severely impaired individuals.

As noted earlier, corrective responses were initiated while the individual maintained their hand at the central location. This ensured differences in baseline behaviour was relatively similar for healthy controls and individuals with stroke. However, some participants with stroke had difficulties maintaining their hands at the start position, leading to impairment in Endpoint Distance for No Shift trials (LA, *n* = 4; RA, *n* = 3). This difficulty to maintain the hand at the central location may have impacted how the mechanical load moved the hand or altered the distance from the hand’s starting position to intercept the target. However, the low hand speed means that the expected effect on motor corrections should be minimal.

## Conclusions

The present study describes the implementation of a novel interception task designed to quantify rapid feedback responses in a population of individuals with stroke. We were able to quantify sensory processing impairments in most individuals with stroke in both their affected and unaffected arms and that these impairments could be specific to one sensory system. This study highlights the prevalence of impairments in generating rapid feedback responses, a skill that is likely important for performing many daily activities and reducing the risk of falls.

## Data Availability

The data used for this study are available upon request from the corresponding author. Clinical data are not available to the public as the information could compromise the privacy of the participants with stroke.

## Abbreviations

BITC: Behavioral inattention test conventional subtest
CI: Confidence interval
CMSA: Chedoke McMaster Stroke Assessment
CMSAa: Chedoke McMaster Stroke Assessment Stage of arm
CMSAh: Chedoke McMaster Stroke Assessment Stage of hand
FFIT: Fast Feedback Interception Task
FIM: Functional Independence Measure
FIM-M: Functional Independence Measure Motor Score
K-S: Kolmogorov-Smirnoff
LA: Left affected
MoCA: Montreal Cognitive Assessment
RA: Right affected
